# Extraction and Determination of Polar Bioactive Compounds from Alfalfa (*Medicago sativa* L.) Using Supercritical Techniques

**DOI:** 10.3390/molecules24244608

**Published:** 2019-12-16

**Authors:** Olga Wrona, Katarzyna Rafińska, Justyna Walczak-Skierska, Cezary Możeński, Bogusław Buszewski

**Affiliations:** 1 Łukasiewicz Research Network–New Chemical Syntheses Institute, 24–110 Puławy, Poland; olga.wrona@ins.pulawy.pl (O.W.); cezary.mozenski@ins.pulawy.pl (C.M.); 2 Department of Environmental Chemistry and Bioanalytics, Faculty of Chemistry, Nicolaus Copernicus University, 87–100 Toruń, Poland; katrafinska@gmail.com (K.R.); walczak-justyna@wp.pl (J.W.-S.); 3 Interdisciplinary Centre of Modern Technologies, Nicolaus Copernicus University, 87–100 Toruń, Poland

**Keywords:** supercritical fluid extraction, SFE, supercritical fluid chromatography, SFC, bioactive compounds, *Medicago sativa* L., response surface methodology, RSM, quarter-technical plant, flavonoids, phenolics compounds

## Abstract

The aim of this research was to select parameters for supercritical extraction with CO_2_ of *Medicago sativa* L., considered as functional food, in quarter-technical plant, providing the highest concentration of bioactive polar constituents and simultaneously maintaining the highest efficiency of the process. For the purpose of optimization, mathematical statistics was used. Qualitative analysis of products was performed with supercritical fluid chromatography (SFC). The SFC analysis revealed a proper separation of flavonoids and phenolics acids for dedicated TFC and TPC optimal parameters. The obtained results have proved that it is a possibility to extract polar compounds with non-polar solvent under higher values of pressure and temperature and to enrich product with desired group of bioactive compounds with proper optimization. The proposed extraction technique allows to obtain on an industrial scale, using an environmentally friendly solvent, a preparation rich in biologically active nutrients that can be implemented in the cosmetics, pharmaceutical and food industries.

## 1. Introduction

Alfalfa (lucerne, *Mediacago sativa* L.) belongs to plant species widely spread in Poland. It is often called “the queen of forages”, mainly because it has been used as inexpensive source of protein in food industry, especially as fodder plant [1]. High interest in the cultivation of the lucerne (in 2016, only in the United States, over 58 Mt of alfalfa was produced) is caused by not only the high content of the protein, but also of the bioactive components in both, aerial parts and roots of this plant. Alfalfa has been considered as functional food due to low content of saturated fat, cholesterol and sodium and high concentration of different nutrients (like folate). Lucerne is also rich in bioactive compounds like phenolic components, saponins (hederagenin, soyasapogenol), essential amino acids (valine, leucine, threonine and lysine), chlorophylls, vitamins (A, E, C, B1, B2, B6 and B12) or β-carotene. It also contains minerals such as Mg, Ca, Cu, Zn, Fe, Mn, P. Due to high content of bioactive nutrients, preparations from alfalfa have antifungal, antibacterial, insecticidal and nematicidal properties [1,2,3,4]. Wherethrough the great content of the bioactive compounds, the alfalfa product has been used as a food and feed additives and medicine supplements. It is typically consumed by humans as an herbal supplement or in the form of alfalfa sprouts. However, a new dimension for application of lucerne products can be added thanks to supercritical extraction by obtaining a pure extract enriched with bioactive nutrients.

Phenols are valuable components in human diet and serve an important role in nutraceutical potential and health-promoting effects due to their high antioxidant activity. Studies have shown that product obtained by extraction of *Medicago sativa* contain various concentration of glycosides apigenin, luteolin, chrysoeriol, tricin, naringenin and methyltricetin [1,5]. As a result of the extraction of lucerne leaves with ethanol as a solvent, genistin, daidzein, glycitein, genistein and formononetin were also determined [6].

The extraction method has a huge impact on the composition of the final extract, including the concentration of polar constituents, like flavonoids [1,7,8,9]. One of the main aims of the extraction is to obtain the highest amount of the extract, which is enriched with desired groups of compounds. This is possible by choosing the suitable extraction method and optimizing the proper process parameters [7]. The most described techniques for extraction of polar bioactive compounds from plant materials are conventional solvent methods: Soxhlet (SE), reflux, maceration or percolation. However, the most popular solvents are methanol, ethanol and their mixtures with water [2,10,11,12,13,14,15]. Furthermore, faster methods like ultrasound assisted extraction (UAE), accelerated solvent extraction (ASE), microwave assisted extraction (MAE) or solid phase extraction (SPE) and matrix solid phase dispersion (MSPD) with polar solvents were also developed in separation of flavonoids from various plant materials [7,9,14,16].

The supercritical fluid extraction as a separation technique is very suitable for the separation according to “green chemistry” standards, especially when carbon dioxide is used as a solvent. The popularity of these techniques (proved by significant number of publications) is connected with the nature of CO_2_ and specificity of the obtained product. Carbon dioxide has a lot of advantages i.e., it is inexpensive, easily available and GRAS–Generally Regarded As Safe [17]. It has interesting physicochemical properties: it is an inert gas, non-polar, tasteless, and odorless and therefore, it does not contaminate the final product. There is also no need to evaporate the solvent, as after the end of process it changes into gaseous state and leaves the process environment itself. As an additional advantage of SFE, all the devices that constitute the plant are hermetic, so the process is carried out without oxygen access which protects bioactive compounds against oxidation [18]. Carbon dioxide reaches critical state at low values of parameters, at 304.4 K (31.1 °C) and the pressure of 7.4 MPa (73.8 bar). Its low critical temperature allows extraction of thermolabile compounds without their prior thermal decomposition [17,18]. Due to the advantages of scCO_2_, SFE has been commercially used in the production of food and feed additives or nutraceuticals and pharmaceuticals. The most common described SFE procedures, applied in the industry, are coffee decaffeination, extraction of hops or hemps, preparation of *Serenoa repens* extract, flower extracts, or natural pesticides [19]. Recent studies have been focused on the selective SFE separation of different groups of compounds from plant materials like waxes from vegetable matters [19,20]. However, scCO_2_ is a hydrophobic and non-polar solvent. The CO_2_ molecule has a small polarity but due to the presence of a quadrupole moment, it can dissolve some polar compounds at higher values of the process parameters [18]. The extractive properties of the supercritical carbon dioxide can be manipulated by adding a defined amount of a polar co-solvent during the process. The role of co-solvent is to increase the polarity and the elution strength of a supercritical fluid with maintaining the same process parameters without significantly changing the scCO_2_ selectivity [17,18]. Wherethrough the SFE separation of polar plant material constituents can be significantly increased.

The SFE extraction of plant materials which leads to obtain product rich in polar components is mostly carried out with polar co-solvent, usually methanol or ethanol solutions [21,22,23,24]. This approach imposes certain limitations. The co-solvent application provides the necessity of the solvent evaporation. On the industrial scale, removal of large amounts of solvent is troublesome because it introduces a new stage that requires specialized equipment and energy inputs.

Therefore, SFE with pure scCO_2_ may seem to be a great separation technique for polar compounds under higher conditions than mostly investigated. So far, there has been no comprehensive investigation about the extraction of polar compounds with supercritical fluid extraction, especially by using carbon dioxide in a supercritical state as a solvent. This is caused mainly by the fact that supercritical carbon dioxide is a non-polar solvent. However, there is a possibility that under proper condition (high value of temperature and pressure) scCO_2_ can sufficiently dissolve polar components. Firstly, due to the presence of a quadrupole moment in the CO_2_ molecule. Secondly, under suitable conditions, the high value of the vapour pressure of polar solute may have a significant influence on the process and it determines extractive properties and efficiency [17,18]. However, due to limitations of most applied devices, there are no investigations where extractions were conducted under range of pressure over 550 bars. In our study, the range of pressure up to 800 bars was investigated.

For qualitative determination of polar compounds in SFE extract, the supercritical fluid chromatography (SFC) was applied. This approach is quite new and not well-described in literature. The advantages of scCO_2_ were described earlier in this article. The same capabilities are found in the SFC separation technique, where scCO_2_ has been used as one of the constituents which makes up the mobile phase. SFC is considered as a technique with “liquid-like solvation power”, “gas-like viscosity” and “tunable solvent” capability [25]. SFC is a green alternative for conventional chromatography with benefits like high efficiency, low organic solvent consumption, and great separation of isomers [25].

The aim of this work was to optimize conditions for extraction of phenolics compounds from *Medicago sativa* L. by pure supercritical carbon dioxide as a solvent. In our study we have assumed that due to the high pressure and temperature of the process, the extraction of polar constituents by non-polar solvent may be controlled by a diffusion mechanism of bioactive substances from the internal parts of the plant matrix. Due to the high value of pressure, the surface tension on the phase boundary can be negligible and the extraction of phenols and flavonoids may be efficient despite of the small solubility in scCO_2_. To take into account the effect of high temperature on the quality of plant material, the level of bioactive compounds in the extracts obtained as a result of maceration carried out at different temperature values was compared. This experiment also allowed to compare the effectiveness of both extraction techniques in obtaining a product enriched in flavonoids and phenolic compounds.

## 2. Materials and Methods

### 2.1. Chemicals and Reagents

Pure (min. 99.9% *v*/*v*) liquid carbon dioxide was produced in Grupa Azoty Zakłady Azotowe “Puławy” PLC. All other chemicals and reagents were purchased from Sigma Aldrich (Steinheim, Germany) and were of analytical grade.

### 2.2. Plant Material

**Figure 1:**
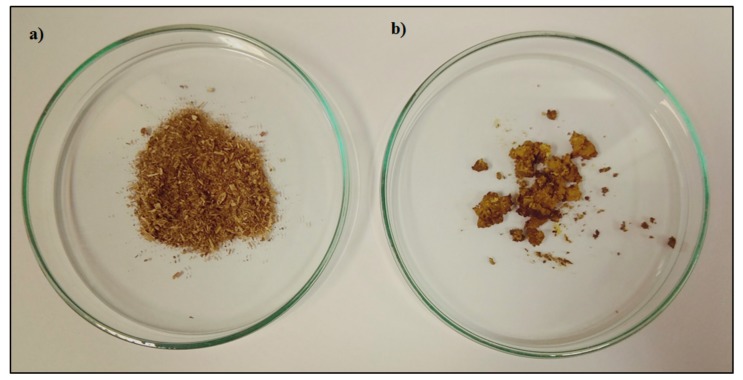
*Medicago sativa* L. as a feedstock (**a**) and obtained extract (**b**).

*Medicago sativa* L. used in this study was harvested in Zalesie, Poland. Alfalfa were dried and ground into fine powder—particle size 1–1.6 mm (Figure 1a). Moisture content of the plant material was 12.1% (*w*/*w*).

### 2.3. Extractions Plan According to Box-Behnken Design (BBD)

BBD was used to verify the effects of various factors: temperature–T/A (313.15–353.15K, 40–80 °C), pressure–P/B (range up to 20–80 MPa), and flow rate of CO_2_–F/C (3–7 kg h^−1^) on the selected criteria. The complete design comprises fifteen experimental steps at the different process conditions (Table 1). To evaluate the effect of those variables the extraction yield (Y, % DM–dry mass), total phenolics content (TPC, mg GAE/g DM), total flavonoids content (TFC, mg RE/g DM) were determined. All the results and statistical analysis were accomplished using Design Expert 11.0 (Stat-Ease, Inc., Minneapolis, MN, USA). Optimal extraction conditions were determined by analysis of variance (ANOVA) based on the obtained responses [26,27].

### 2.4. Supercritical Fluid Extraction of M. sativa at Quarter-Technical Plant

**Figure 2:**
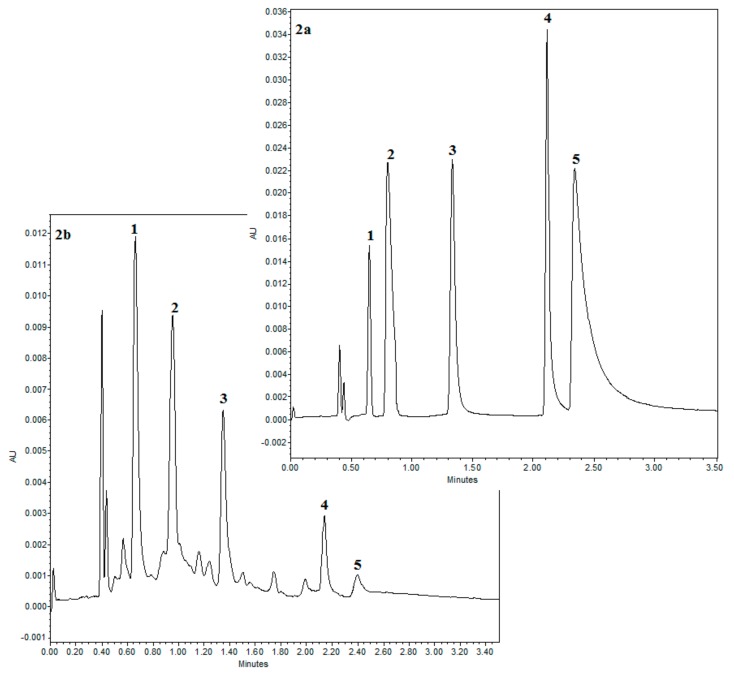
(**a**) Supercritical fluid chromatography (SFC) chromatogram of flavonoids and phenolic acids standards (1–flavon, 2–salicylic acid, 3–ferulic acid, 4–naringenin, 5–apigenin). (**b**) SFC chromatogram of *Medicago sativa* L. plant extract.

Supercritical fluid extraction was performed using quarter-technical plant (SITEC-Sieber Engineering AG, Switzerland) placed in New Chemical Syntheses Institute in Puławy, Poland (Figure 2a). The installation can work under the pressure ranging up to 100 MPa, and the temperature up to 473.15 K (200 °C). 200 g (M = 0.2 kg) of ground dried plant material was loaded into the 1 L extraction vessel. The extraction process was carried out according to well-developed procedure (Wrona et al., 2019). After achieving the required extraction parameters, the supercritical CO_2_ was passed through to the extractor. After specified time, the system was switched to work on the bypass (omitting the extractor vessel) to wash off the deposits remaining in the pipelines. The pressure was reduced to the condensation pressure and the pump was turned off. After proper shutdown of the installation, i.e., the values of process parameters were equal to the environmental ones, the post-extraction residue was removed from the extraction vessel. Finally, the extract accumulated in the separator was carefully collected and analyzed.

### 2.5. Maceration

One gram of ground dried *M. sativa* was soaked in 10 mL of 96% ethanol solution for 3 h at 40, 60 and 80 °C (313.15, 333.15 and 353.15 K respectively) in the dark and it was incubated in water bath with stirring (Julabo SW, 22 Seelbach, Germany). After this time, extracts were centrifuged (4000× *g*) (Eppendorf™ Centrifuge 5810R, Hamburg, Germany), and the clear supernatant was collected. The obtained extracts were analyzed according to described procedures.

### 2.6. Total Phenolics Content (TPC)

The Folin-Ciocalteau (F-C) method was used for determination of the total phenolics content where100 µL of sample were added to 1.5 mL deionized water and 100 μL F-C phenol reagent. After 8 min, 300 μL of 20% sodium carbonate was added to the mixture. Absorbance spectra were recorded at 765 nm against a blank after 30 min at 20 °C in the dark. In blank solution, 100 µL of extract was replaced with 100 µl of 96% ethanol solution. Results were expressed as mg gallic acid equivalents (GAE)/g DM.

### 2.7. Total Flavonoids Content (TFC)

The total flavonoids content (TFC) was calorimetrically determined by using aluminum chloride (AlCl3). The 0.25 mL of sample was mixed with 0.5 mL of 2% AlCl3 (in 96% ethanol solution) and diluted to a specified volume (1 mL) with EtOH. The incubation time of the sample kept at ambient temperature was 40 min. Absorbance was measured against a blank (0.25 mL of sample and 0.75 mL of 96% ethanol) at 420 nm. TFC were expressed as mg rutin equivalents (RE) using a calibration curve from rutin solutions in 96% ethanol solution (EtOH).

### 2.8. Supercritical Fluid Chromatography

SFC analysis was performed using an Acquity UPC2 system (Waters, Milford, MA) with a binary solvent pump, a column oven, an autosampler, PDA detector and backpressure regulator (BPR). Methanol was used as the needle wash solvent. Data acquisition and control of UPC2 were carried our using the Waters Empower 3 software. The SFC final analysis was performed at 25 °C on ACQUITY UPC BEH column (100 × 3 mm, 1.7 μm, Waters, Milford, MA). The elution gradient (eluent A: CO_2_/B: MeOH) involved: 0-6 min, 95–65% A, 6–8 min, 65–65% A, 8–10 min, 65–90% A. The backpressure was set to 13.7 MPa, the flow rate was 1.5 mL/min, and the injection volume was 4 µL. The PDA detection wavelength was set at 254 nm.

### 2.9. Preparation of Standards and Samples Solutions for SFC Determination

All standards were dissolved in methanol to prepare 2 mg/mL of stock solutions. The stock solutions were diluted with methanol from 0.001 to 1.0 mg/mL to obtain calibration curves. Each sample was injected three times. All standard solutions were stored at 4 °C until use and filtered through a 0.22 µm membrane before the injection. The method was validated. The validation parameters included the linearity, correlation coefficient (r^2^), LOD and LOQ. LOD and LOQ at signal to noise ratio were three (S/N = 3) and ten (S/N = 10) times to noise level, respectively.

## 3. Results and Discussion

### 3.1. SFE-scCO_2_–Result of the Optimization

In this paper, extraction with supercritical carbon dioxide of alfalfa was performed. Extraction of the entire dried and ground plants allow to obtain the whole variety of bioactive nutrients, which are normally placed in different part of plant. The new approach of this study was to extract non-oleic plant with pure scCO_2_ to obtain product with high efficiency and high level of bioactive compounds as phenolics compounds. Polar compounds are not efficiency extracted by carbon dioxide. In order to induce changes in the solubility or diffusivity of those compounds, high process parameters, particularly pressure and temperature should be used. This approach is impossible with common laboratory equipment, because the upper pressure range in those devices is approximately 500 bar [16,21,22,23,26]. Furthermore, on a laboratory scale, the process is carried out with the use of a small amount of feedstock, which means that without dissolving the product in additional co-solvent, it would not be possible to collect the extract from the separator. In the case of our work, we have worked at the quarter-technical plant where the amount of the feedstock and obtained product allows work with pure supercritical fluid. We took advantage of the available SFE installation. We applied pressures up to 80 MPa which allows studies of the extractability of polar compounds in the extensive range of process parameters. We also used high values of temperature up to 80 °C to check the temperature effect on the content of polar compounds (Table 2). In order to verify the influence of the temperature on the concentration of polar compounds, conventional macerations with 96% ethanol solution at defined in BBD plan values of temperature, were performed. An increase in temperature up to 80 °C was associated with an increase in the content of phenolic compounds and a decrease in the content of flavonoids. The total content of phenolic compounds and flavonoids in the extract obtained in 80 °C was almost twice as high as in the extract obtained in 40 °C, which clearly indicates that high temperature does not adversely affect the content of bioactive compounds. Maceration with polar co-solvents is well described and has been dedicated for polar compounds separation [10].

As it was mentioned earlier in this paper, pure scCO_2_ was used as a solvent and obtained extract was in the form of dense yellow-green paste (Figure 1b). For this reason, there was no need to evaporate the sample to dry mass. Proper amount of extract was measured, diluted in specific solvent and destined for further analysis. All of the obtained results are summarized in the Table 1. The process parameters are presented in both: coded and uncoded forms. The values of process efficiency were in the range 1.27–6.72% DM. The total content of phenolics was from 5.32 to 28.08 mg GAE/g DM. The maximum value for total flavonoids content (13.16 mg RU/g DM) was obtained for experiments no. 1 (E1) where medium temperature and the highest value of pressure and solvent flow rate were implemented.

Analysis of TPC and TFC level in extracts obtained by macerations at different T values confirmed that it cannot be strictly concluded that temperature causes the decrease in the concentration of polar constituents. The amount of TPC increases with temperature rising, up to 28.16mg GAE/mL for experiments conducted under the highest value of temperature. Temperature has different impact for the TFC content: firstly the concentration of flavonoids increases with temperature, and finally it reaches the lowest value at 353.15 K (80 °C). This may indicate a thermal decomposition of some extracted flavonoids. According to Box-Behnken plan data for the SFE experiments (Table 1), for extract no. 1 obtained at 60 °C the determined value of TFC was also the highest. Summarizing both the determined amounts of TPC and TFC for the same temperature, it can be observed that the concentrations of analyzed groups of constituents in extract obtained under 353.15 K (80°) is the highest, 31.42 mg/mL. Considering the results of maceration and supercritical fluid extraction for the highest tested temperature (80 °C), we can conclude that flavonoids are more susceptible for thermal decomposition than phenolics.

The empirical second-order polynomial equations (in coded and uncoded forms), coefficient of determination (R^2^), model F-value, lack of fit F-value and *p* value of model which represent all the selected responses are summarized in the Table 3. Those crucial statistical values exhibit a proper fit in analyzed range of output values which indicate the proper model fitting. Coefficient of determination as the most important value, provides information about proper fitting between output and input variables and provides an estimate of the strength of the relationship between the obtained model and the response variable. R^2^ for yield was 0.94 which has proven the correctness of the adopted model. Non-statistically significant *lack of fit F*-value (4.31 > 0.05) and statistically significant *p*-value (0.0135 < 0.05) indicate that the model describes correctly the dependence of the response and process parameters. For yield, the Model F-value of 8.87 also implies the fitting is significant. The results for TPC and TFC can be interpreted by analogy.

For all responses, in the examined range of variability of T, P and F, the adopted models described properly the dependence of input and output variables. However, various parameters were statistically significant in the case of individual response. Statistically significant model terms for individual responses were:

- for yield–temperature and solvent flow rate,
- for TPC–temperature, pressure, solvent flow rate, linear correlation between pressure and flow rate,
- for TFC–pressure, solvent flow rate, linear correlation between temperature and pressure and squared pressure. It can be noticed that pressure has a crucial impact on the TFC content.

This section may be divided by subheadings. It should provide a concise and precise description of the experimental results, their interpretation as well as the experimental conclusions that can be drawn.

Optimized elution strength of supercritical fluid (SF) and the solute solubility in the SF have a crucial impact on the extraction efficiency. Those factors strictly depend on the values of extraction temperature and pressure. T and P have a direct impact on the density of SF, where small changes in those parameters may cause sizable changes of extractive properties. Density of SF decreases but the vapor pressure of solute increases with the increasing temperature. With the increasing pressure, both of those factors also increase. Moreover, the scCO_2_ density decreases with increasing temperature, so the extraction of non-polar compounds decreases too. These changes improve the selectivity of the extraction of polar constituents. Finally, the diffusivity in supercritical carbon dioxide and thermal conductivity increases with increasing temperature [17,18]. Concluded, values of temperature and pressure have a decisive influence on the yield of the extraction and the composition of the final product. Proper solvent flow rate provides a great mass transfer, because it has an impact on differences in concentration of the solute dissolved in the solvent and the solute remaining in the plant materials.

The influence of three chosen input variables: temperature (T/A), pressure (P/B) and solvent flow rate (F/C) and their interactions were determined and presented as response surface plots in Table 4. The bigger slope indicates the stronger dependence as the evaluation index increases faster. Based on the obtained response surface plots, it can be concluded that the interactions between all variables have the effect on all responses. The yield of the process increases with the increase of temperature and solvent flow rate (which also has been proven by the regression equations, where significant model terms are T and F). The pressure effect on the extraction efficiency is lower but more complex. Firstly, the yield of the process increases with pressure, but after achieving maximum, it decreases. Total phenolics content increases with pressure and decreases with the increase of solvent flow rate. The value of temperature has a complex impact on the extraction of phenolics constituents because the highest and the lowest value of determined group of bioactive compounds was obtained for the same T value. Due to the quite interesting response surface plots were obtained for that response. The correlation of all input factors is crucial, and all of the input variables have impact of the maximum TPC content (which is confirmed by significance of all T, P and F in polynomial equation). The strongest influence of three input variables, especially pressure is observed for total flavonoids content, which is indicated by the variety of colors forming the plots surface as well as by the variety of the significant model terms in the obtained regression equations. The highest amount of TFC was obtained for high values of T, P and F.

The optimal conditions, obtained as a result of extraction of lucerne at quarter-technical plant, and analyzed data by RSM and ANOVA were determined and summarized in the Table 5. The main criterion was to achieve the highest content of each response. The highest amount of extract, according to analysis of variance, should be obtained during the extraction under T = 352.99 K (79.80 °C), P = 78.55 MPa and F = 6.83 kg h^−1^. In the process conducted at 353.15 K (80 °C), 71.74 MPa and 3.32 kg h^−1^, the highest amount of TPC should be obtained. The highest concentration of total flavonoids can be achieved in the extraction carried out at high values of all process parameters.

The economics of process is always crucial, especially when the extraction is carried out at larger scale than laboratory (in our study it was quarter-technical plant). To provide the minimum cost of the optimization process, the statistical and mathematical methods were used to optimize the number of experiments. Therefore, the maximal efficiency of the process is the main optimization criterion. But in this study, the optimal conditions obtained for yield and TFC are in the same range. Three of the analyzed input variables for those responses are very high. Therefore, it is possible to optimize the extraction of *Medicago sativa* L. based on following criterion: the highest yield of the process should provide high concentration (under confidence interval) of flavonoids in optimized product.

In order to verify the accuracy of the process modeling, extractions in optimal parameters at quarter-technical plant for all three responses separately were carried out. The predicted value of the yield under optimized parameters should be 7.08 % DM. In our study, after extraction under optimal conditions, it was obtained m = 14.79 g of the product (M = 200g), which indicated the value of 7.39% DM. The result equally confirms the accuracy of the prediction. The total phenolic content in the obtained product under optimal conditions was 20.20 mg GAE/g DM. This value is slightly below the range within the confidence interval. This error does not have to be a result of an incorrect optimization of any steps but it may be related with different, unexpected factors caused by changes in the procedural operation resulting from working on a large scale. The determined concentration of TFC in the extract obtained under 351.51 K, 78.29 MPa and 6.72 kg h^−1^ was slightly lower than the predicted ones, but it is in the range within the confidence interval.

Statistically significant model terms for individual responses provides information about the influence of the particular input variables on selected criteria. This can be observed especially for TFC. Throughout the simple maceration procedure, it was proved that the content of flavonoids was the lowest for extract obtained at 80 °C. However, as a result of ANOVA the optimal conditions for the highest amount of TFC were T = 351.51 K (78.40 °C), P = 78.29 MPa and F = 6.52 kg h^−1^. In this case, the value of optimal temperature was very high, which was not compatible with the results obtained for maceration. However, it cannot be ruled out that various flavonoids with different resistance to high temperatures are isolated during maceration and SFE. Pressure has the strongest impact on the TFC content and the value of optimal pressure determine the optimal temperature (which was also confirmed by the regression equation and the coefficients in equation). Additionally, the final results also confirm the necessity to study the influence of more than one input variables on a chosen response as well as their interactions.

In the extract, obtained under optimal conditions for the maximum yield of the process, the total flavonoids content was analyzed. The goal of this analysis was to verify the possibilities of optimization simultaneously with two different criteria. The obtained TFC content was 14.56 mg RU/g DM which was under optimal range. This data proved the accuracy of the process optimization with individual criteria separately, as well as the according to different criteria at the same time. However only when the values of optimal parameters are to be in the similar range. Finally, it can be concluded that the adopted optimization procedure can be used to predict the studied values in the accepted range of variability of input factors.

The obtained results indicate that the extraction efficiency of polar bioactive compounds from *M. sativa* with supercritical carbon dioxide is comparable to conventional extraction techniques. SFE was especially effective in isolation flavonoids compared to maceration (14.89 mg RU/g DM for SFE and 10.34 mg RU/g DM for maceration at 60 °C). However, the total content of phenolic compounds was lower in extract obtained during SFE (20.20 mg GAE/g DM) compared to maceration with 96% ethanol at 80°C (26.16 mg GAE/g DM).

Similar correlation was observed for extraction of *Lepidium sativum* freeze-dried sprouts and seeds with different techniques. The highest level of flavonoids was noticed for SFE with 96% ethanol as co-solvent–it was over 6 times as high as for maceration with 70% ethanol [28]. The effectiveness of this technique in the extraction of flavonoids from plant material was also confirmed by many other studies, for example on *Maydis stigma*, *Scutellaria baicalensis* and *Ginkgo* leaves [29].

### 3.2. Result of SFC Analysis

SFC is an orthogonal technique to reserved phase HPLC, because the retention mechanism is similar to normal phase HPLC. Therefore, the selection of stationary phase is decisive when developing an analytical method. In order to separate each flavonoids and phenolic acids, the chromatographic conditions were optimized. The analyzed compounds were separated with good resolution and peak shapes in a short time analysis (3 min). In the research, was tested bridged ethyl hybrid (BEH) stationary phase. The quantitative and qualitative analysis was performed under the optimized conditions at 25 °C and 13.7 MPa using methanol as the co-solvent (the gradient as described in Section 2.8). Figure 2a shows the separation of references substances which eluted in the following order: flavon, salicylic acid, ferulic acid, naringenin, and apigenin. In case of naringenin and apigenin, were observed strongest retention, which is probably results of π–π interactions and hydrogen bonding with proton-donors between aromatic moieties from flavonoids and polar stationary phase [30]. This affects the band broadening during the chromatographic separation and also affects mass transfer, which includes thermodynamic processes responsible for system selectivity [31].

The method was validated and all results are compiled in Table 6. The calibration curve for each compound was linear over the concentration range presented in Table 6. The satisfactory results were obtained for the five compounds tested, including correlation coefficient higher (r^2^) than 0.9985. The LODs were between 1.00 µg/g and 20 µg/g while LOQs were from 3.3 µg/g to 66 µg/g which indicated good linearity and sensitivity.

In the study, the method of analyzing three flavonoids (flavon, apigenin, naringenin) and two phenolic acids (salicylic and ferulic acid) present in Yield, TPC and TFC extracts from *M. sativa* was described. The concentrations of flavon, salicylic acid, ferulic acid, naringenin and apigenin were determined for extracts from *M. sativa* obtained under optimized conditions and they are listed in Table 7. All analyses were carried out in triplicate. The concentration of flavon is the highest in extract optimized for TFC, and concentration of naringenin and apigenin is also higher for this extract. The lowest level among the tested phenolic compounds was observed for ferulic acid.

It is worth noting that the flavonoids content was higher in the sample obtained under optimal TFC conditions specified for that group of compounds. The same correlation is observed for extract obtain under for conditions optimal for TPC, where the content of salicylic and ferulic acids is the highest (Figure 2b).

## 4. Conclusions

The goal of this study was to optimize parameters for scCO_2_ extraction of alfalfa in quarter-technical plant providing the highest yield of the process as well as the highest amount of phenolics and flavonoids compounds in the extract. All of the investigated extraction parameters i.e., temperature, pressure and flow rate had an influence on yield, total phenolics content and total flavonoids content. The highest yield, content of TPC and TFC were determined for extracts obtained under 352.99 K, 78.55 MPa, 6.83 kg h^−1^; 353.15K, 71.74 MPa 3.32 kg h^−1^ and 351.51 K, 78.29 MPa, 6.72 kg h^−1^, respectively. All of the three optimal parameters providing the highest yield and TFC were in the similar range. These results revealed that pure supercritical carbon dioxide despite its non-polar character can be efficient in isolation of phenolics and flavonoids but high values of parameters such as temperature and pressure are required. The results confirm that the separation of polar constituents by non-polar scCO_2_ is efficiently determined by the diffusion where mass transfer is controlled by convection. The SFC analysis of extracts obtained under optimal conditions for TFC and TPC revealed that the extracts have higher content of individuals group of flavonoids and phenolic acids, respectively. All the obtained data have proved that the optimization of supercritical carbon dioxide extraction of *Medicago sativa* L. at quarter-technical plant has ended successfully and that it is possible to extract polar compounds with non-polar solvent under higher values of pressure and temperature and enriched product in desired group of bioactive compounds with proper optimization. The obtained extraction residue can be used as feedstock and the extract, enriched in bioactive compounds, can be implemented as a functional food additive and human diet supplement.

## Figures and Tables

**Table 1:** Responses obtained by realizing Box-Behnken plan for the SFE-scCO_2_ of lucerne at quarter-technical plant.

	Box-Behnken Design			Results		
	T, *K*	P, *MPa*	F, *kg h^−1^*	Y, *% DM*	TPC, *mg GAE/g DM*	TFC, *mg RU/g DM*
E1	333.15 (0)	80.00 (1)	7.00 (1)	4.53	6.32	13.16
E2	353.15 (1)	80.00 (1)	5.00 (0)	5.76	11.86	11.42
E3	353.15 (1)	20.00 (−1)	5.00 (0)	3.47	5.38	0.00
E4	313.15 (−1)	50.00 (0)	3.00 (−1)	1.27	26.19	2.11
E5	333.15 (0)	50.00 (0)	5.00 (0)	4.19	9.65	8.96
E6	353.15 (1)	50.00 (0)	3.00 (−1)	4.58	17.00	7.13
E7	333.15 (0)	50.00 (0)	5.00 (0)	3.50	11.58	8.37
E8	333.15 (0)	50.00 (0)	5.00 (0)	3.67	14.42	11.19
E9	313.15 (−1)	80.00 (1)	5.00 (0)	2.03	18.97	0.32
E10	333.15 (0)	20.00 (−1)	3.00 (−1)	3.39	7.83	0.36
E11	313.15 (−1)	20.00 (−1)	5.00 (0)	1.47	19.01	8.96
E12	313.15 (−1)	50.00 (0)	7.00 (1)	2.72	17.59	12.15
E13	333.15 (0)	80.00 (1)	3.00 (−1)	1.89	28.08	3.41
E14	333.15 (0)	20.00 (−1)	7.00 (1)	3.31	9.01	0.36
E15	353.15 (1)	50.00 (0)	7.00 (1)	6.72	7.87	10.86

**Table 2:** Results of total phenolics content (TPC) and total flavonoids content (TFC) in *M. sativa* extracts obtained by maceration.

T, *K*	TPC, *mg GAE/mL*	TFC, *mg RU/mL*	Σ_TPC + TFC_, *mg/mL*
313.15	8.51	8.49	17.00
333.15	10.98	10.34	21.32
353.15	28.16	3.26	31.42

**Table 3:** Polynominal quadratic equations for all output variables as results of the studies on the *Medicago sativa’s* extracts.

Output Variables		Regression Equations (Significant Model Terms Are Colored)	Coefficient of Determination, R^2^	Model F-Value	*Lack of Fit F*-Value, LOF	*p* Value of the Model
Y, % DM	coded	Y = 3.7744 + 1.6305A + 0.3180 B + 0.7674 C + 0.4325 AB + 0.1725 AC + 0.68 BC − 0.0308 A^2^ − 0.5733 B^2^ + 0.0647 C^2^	0.94	8.87	4.31	0.0135
	uncoded	Y = −13.5295 + 0.0753 T − 0.2224 P − 1.7857 F + 0.00072 TP + 0.0043 TS + 0.0113 PF–0.000077 T^2^ − 0.0006 P^2^ + 0.017 F^2^				
TPC, mg GAE/g DM	coded	TPC = 11.8833 − 4.9563 A + 3 B − 4.7887 C + 1.63 AB − 0.1325 AC − 5.735 BC + 3.1371 A^2^ − 1.2154 B^2^ + 2.1421 C^2^	0.96	12.78	0.98	0.0059
	uncoded	TPC = 997.7188 − 5.5927 T − 0.1921 P − 1.8669 F + 0.0027 TP − 0.0033 TS − 0.09558 PF + 0.0078 T^2^ − 0.00135 P^2^ + 0.5355 F^2^				
TFC, mg RU/g DM	coded	TFC = 9.51 + 0.7337 A + 2.33 B + 2.94 C + 5.02 AB–1.58 AC + 2.44 BC–0.2958 A^2^–4.04 B^2^–1.15 C^2^	0.92	6.20	3.57	0.0293
	uncoded	TFC = −30.68185+0.308521 T–2.46040P + 15.44277F + 0.008358 TP − 0.039437 TF + 0.040625 PF–0.0000740T^2^ − 0.004484 P^2^ − 0.287083F^2^				

R^2^ > 0.8–statistically important, *p* < 0.0001–very highly significant, *p* < 0.01 very significant, *p* < 0.05 significant, *p* > 0.1 not statistically significant. LOF > 0.05–statistically important.

**Table 4:** Response surface plots of the different criteria of *Medicago sativa* L. extract as a function of main process parameters.

Response Surface Methodology–Graphical Results			
	Response Surface Plot as a Function of: Pressure (P) and Temperature (T)	Response Surface Plot as a Function of: Solvent Flow Rate (F) and Temperature (T)	Response Surface Plot as a Function of: Pressure (P) and Solvent Flow Rate (F)
**Y, % DM**	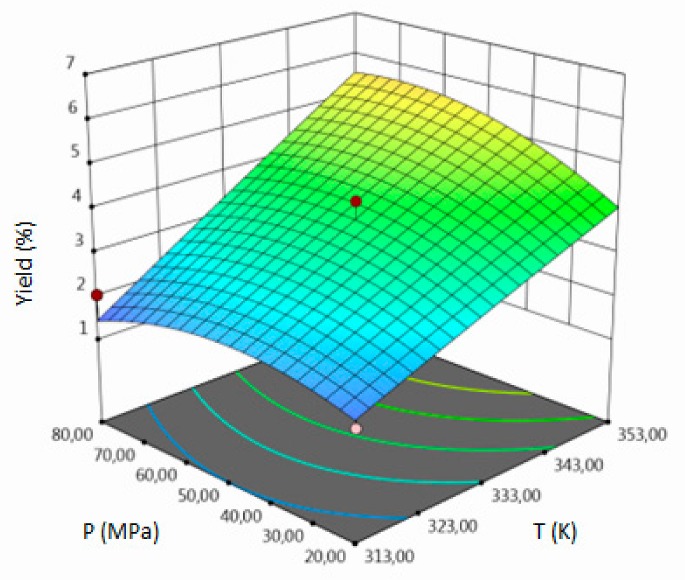	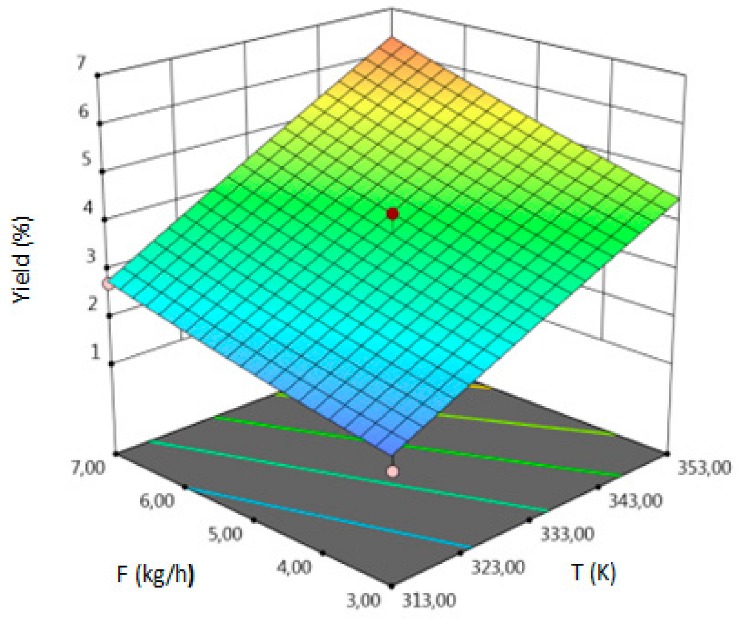	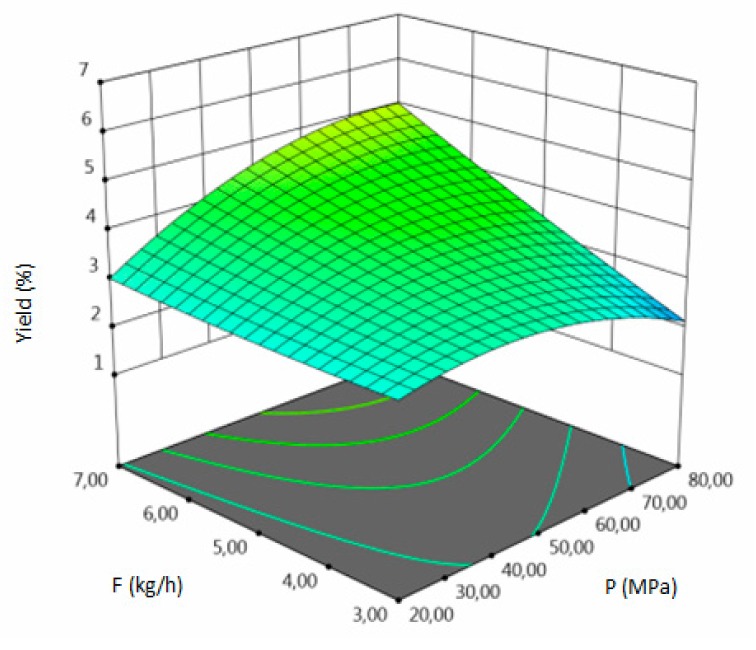
**TPC, mg GAE/g DM**	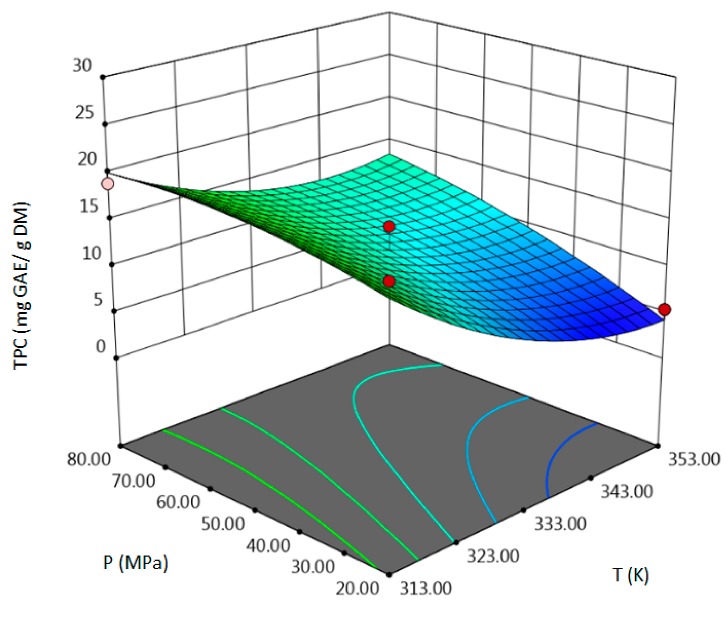	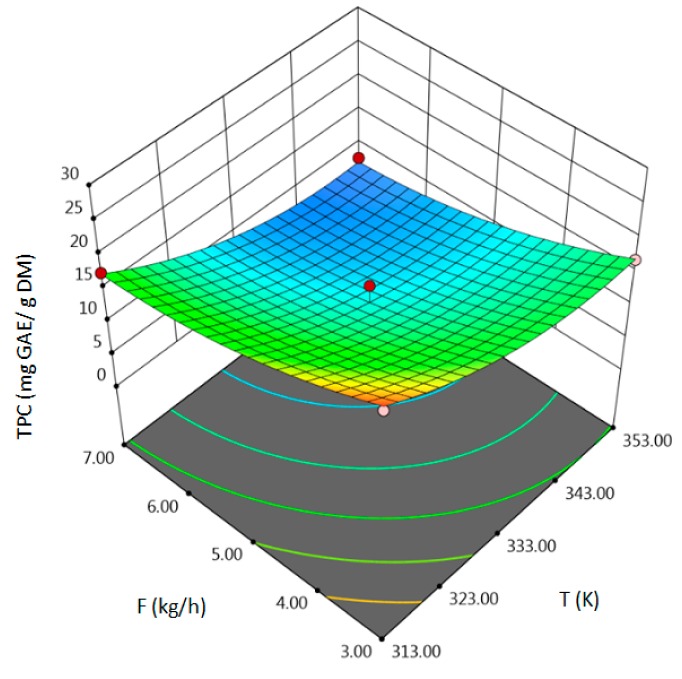	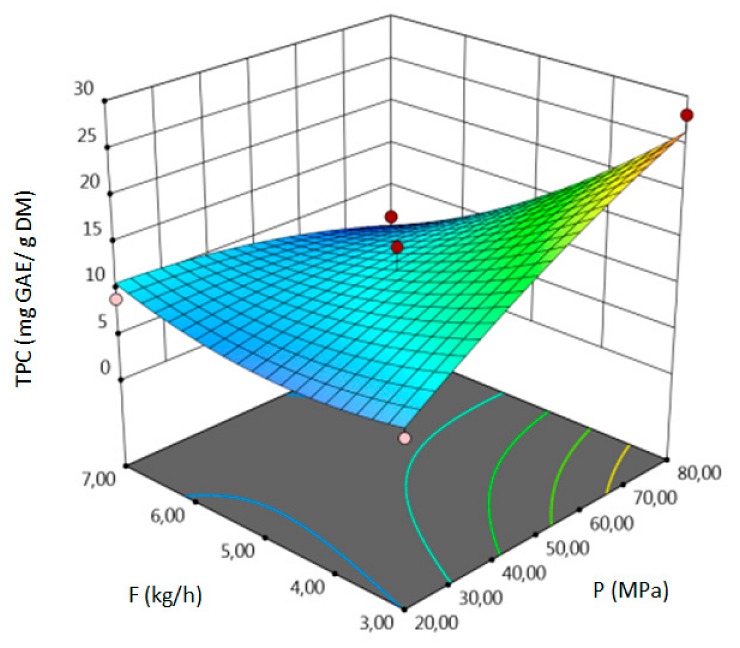
**TFC, mg RU/g DM**	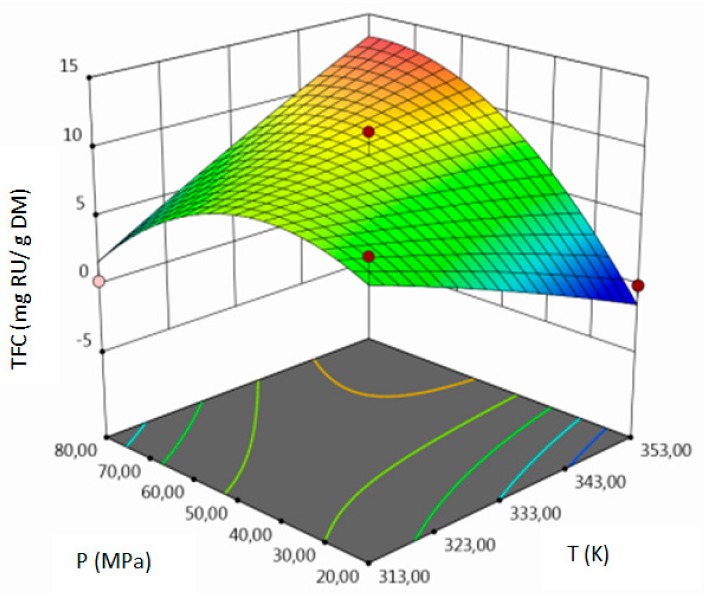	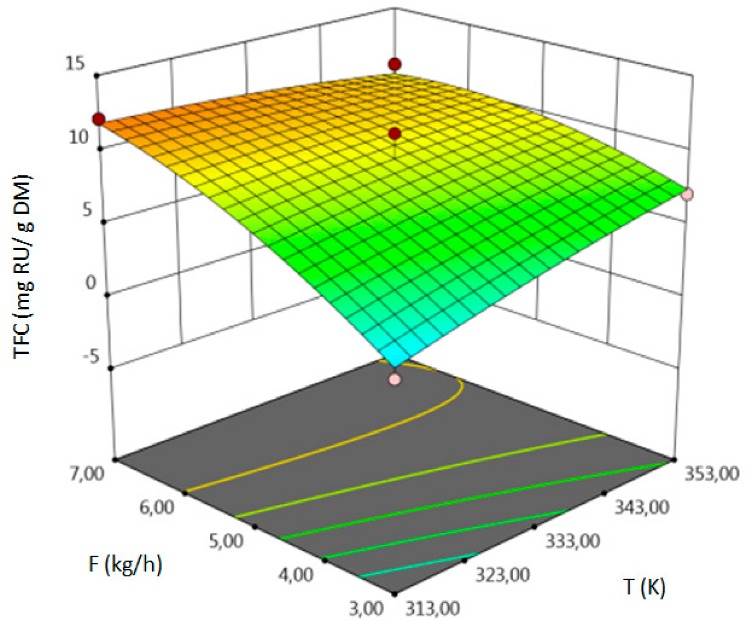	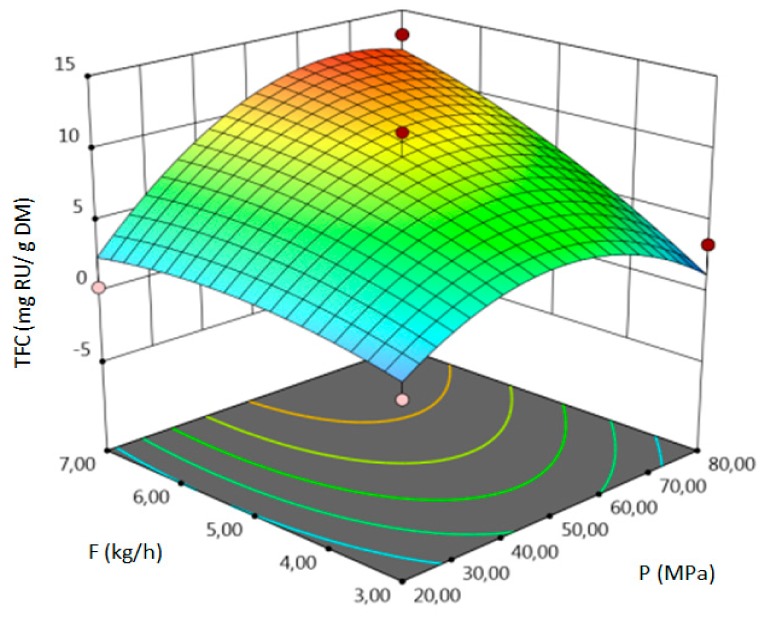

**Table 5:** Results of analysis of variance of obtained values and the comparison of predicted and experimental values of each evaluation index.

Output Variables	Optimal Conditions			Predicted Value	Actual Value	Confidence Interval	
	T, K (°C)	P, MPa	F, kg h^−1^				
	Desirability-1.0					−95%	95%
Yield, % DM	352.99 (79.80)	78.55	6.83	7.08	7.39	6.72	7.42
TPC, mg GAE/g DM	353.15 (80)	71.74	3.32	21.92	20.20	20.82	23.02
TFC, *mg RU/g DM*	351.51 (78.40)	78.29	6.72	15.32	14.89	14.55	16.09

**Table 6:** Basic validation parameters of the flavonoids and phenolic acids determination method using SFC (*n* = 3).

Analytes	Range [mg/mL]	Regression Curve	r^2^	LOD [µg/g]	LOQ [µg/g]
Flavon	0.001–1	y = 1334023x − 3680	0.9998	1	3.3
Salicylic acid	0.05–1	y = 32304x − 1279	0.9992	20	66
Ferulic acid	0.02–1	y = 308257x + 1274	0.9990	10	33
Naringenin	0.02–1	y = 181025x − 4073	0.9990	10	33
Apigenin	0.02–1	y = 505689x − 5334	0.9985	10	33

**Table 7:** Concentration of flavonoids and phenolic acids in extract obtained under optimal condition for Yield, TPC and TFC from *M. sativa*.

Samples	Flavonoids Content μg/g DM (n = 3)			Phenolic Acids Content μg/g DM (n = 3)	
	Flavon	Naringenin	Apigenin	Salicylic Acid	Ferulic Acid
Yield	1.64	35.9	12.3	49.5	1.2
TPC	16.9	37.8	15.0	53.4	4.9
TFC	22.0	41.4	17.9	49.9	4.2

## Abbreviations

ANOVA	analysis of variance
ASE	accelerated solvent extraction
BBD	Box-Behnken design
DM	dry mass/dry matter
F (kg/h^−1^)	solvent flow rate
GAE	gallic acid equivalent
LOF	lack of fit
m (g)	mass of the extract
M (kg)	mass of the feedstock
MAE	microwave solvent extraction
MSPD	matrix solid phase dispersion
P (MPa)	pressure
R^2^	coefficient of determination
RU	rutin equivalent
RSM	response surface methodology
scCO_2_	supercritical carbon dioxide
SE	Soxhlet extraction
SFC	supercritical fluid chromatography
SFE	supercritical fluid extraction
SF	supercritical fluid
SFE-scCO_2_	supercritical fluid extraction - supercritical carbon dioxide
SPE	solid phase extraction
T (K)	temperature
TPC	total phenolics content
USAE	ultrasound assisted extraction
